# Mechanostimulation Protocols for Cardiac Tissue Engineering

**DOI:** 10.1155/2013/918640

**Published:** 2013-07-08

**Authors:** Marco Govoni, Claudio Muscari, Carlo Guarnieri, Emanuele Giordano

**Affiliations:** ^1^BioEngLab, Health Science and Technology-Interdepartmental Center for Industrial Research (HST-CIRI), University of Bologna, I-40064 Ozzano Emilia, Italy; ^2^Department of Biomedical and Neuromotor Sciences (DIBINEM), University of Bologna, I-40126 Bologna, Italy; ^3^Laboratory of Cellular and Molecular Engineering “Silvio Cavalcanti,” Department of Electrical, Electronic, and Information Engineering “G. Marconi” (DEI), University of Bologna, I-47521 Cesena, Italy

## Abstract

Owing to the inability of self-replacement by a damaged myocardium, alternative strategies to heart transplantation have been explored within the last decades and cardiac tissue engineering/regenerative medicine is among the present challenges in biomedical research. Hopefully, several studies witness the constant extension of the toolbox available to engineer a fully functional, contractile, and robust cardiac tissue using different combinations of cells, template bioscaffolds, and biophysical stimuli obtained by the use of specific bioreactors. Mechanical forces influence the growth and shape of every tissue in our body generating changes in intracellular biochemistry and gene expression. That is why bioreactors play a central role in the task of regenerating a complex tissue such as the myocardium. In the last fifteen years a large number of dynamic culture devices have been developed and many results have been collected. The aim of this brief review is to resume in a single streamlined paper the state of the art in this field.

## 1. Introduction

Bioengineered tissue is a potential solution for the replacement of a damaged failing heart [1, 2]. In this respect, the ability to emulate in a cell culture the physical cues involved in the physiological development of a normal cardiac tissue is a key for a successful application of tissue engineering in regenerative medicine. Although bioengineered tissues such as skin [3] and bone [4] are already a clinical option available to patients, cardiac muscle tissue engineering is a present challenge in biomedical research—albeit several studies witness a definite advancement in this field [5].

“Cell therapy,” that is, the direct injection of cell suspensions in damaged cardiac areas has a documented potential for cardiovascular repair [6]. Obviously, which type of cells has to be used to generate an artificial heart tissue represents a relevant issue, since it heavily impacts the final properties of a graft. As a matter of fact, cells should at least be highly viable, able of electromechanical integration with the resident healthy cardiomyocytes, and possibly histocompatible. In this respect, the effectiveness of grafting stem cells (SCs) into a damaged heart is nowadays more than just a proof of principle [7–10]. It is well established that embryonic SCs (ESCs) are able to generate cardiomyocytes [11]. However, ethical and technical issues (risk of teratoma following transplantation of ESC-derived cardiomyocytes [12]) limit their clinical potential. The ability to generate induced pluripotent SCs (iPSCs) [13] provides an approach for the generation of autologous grafts. Moreover iPSCs appear able of cardiomyogenic differentiation [14]. Their main limitations for the clinical use are the time requirement for the reprogramming procedure and, again, the need to ensure that they are nontumorigenic. Resident cardiac SCs are another promising phenotype that can be isolated from identifiable cardiac niches and expanded *ex vivo* [15]. However, the high invasiveness of the withdrawal technique limits their clinical application. Thus, recently adult mesenchymal SCs (MSCs) have been extensively investigated, aiming to their use in biological constructs for cardiac repair, due to the relative ease and safeness of their procurement—mainly from bone marrow and adipose tissue—also in humans. [16–18]. Irrespective of the phenotype, cell therapy is however hampered by the poor survival of injected cells: in fact, most of them die shortly after grafting into the injured heart where hypoxia, nutrient deprivation, loss of survival signals, and inflammation are all responsible for contributing to a hostile environment [19–21]. Engineering a pseudotissue *in vitro* for subsequent engraftment *in vivo* was thus proposed as a more suitable approach than the direct cell injection. In this case, a scaffold should provide a structured environment with tissue-specific mechanical properties and the ability to integrate with surrounding tissue [22, 23]. Biomaterials used for scaffold fabrication include biological molecules (e.g., alginate, collagen, fibrin, and hyaluronan) and biomimetic synthetic polymers (e.g., polylactic and polyglycolic acids and their copolymers, polycaprolactone) where a specific supramolecular architecture is designed to sustain the differentiation and functional organization of the seeded cells [24–31].

**Figure 1:**
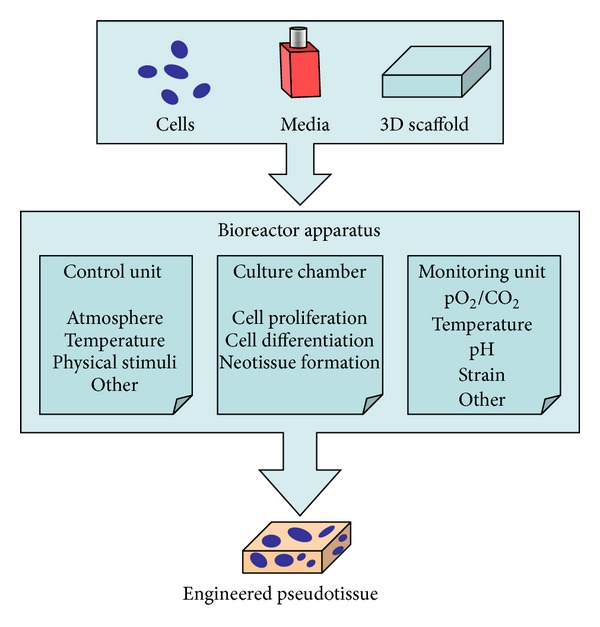
Schematic description of the common features of bioreactors for tissue engineering.

The appropriate elastic modulus and a 3D environment of the scaffold are preferred to drive the differentiation of SCs into cardiac muscle tissue [32–34]. Cell expansion and differentiation in culture, to develop a cardiac pseudotissue *ex vivo*, will take advantage of using a bioreactor, to guarantee environmental conditions and biophysical parameters able to induce, sustain and enhance the development of engineered cardiac graft. Introduction of bioreactors in tissue engineering was driven by the need to apply defined culture regimes [35] and they can be defined as any apparatus able to provide *in vitro* a favorable physicochemical environment to promote physiological conditions for cell/tissue growth. In a general way, they share some common features, such as maintaining the desired concentration of gases and nutrients in the culture medium, establishing a uniform distribution of cells on a 3D scaffold, and exposing the developing tissue to physical stimuli according to the functional requirements of the tissue to be engineered (Figure 1) [36]. 

**Figure 2:**
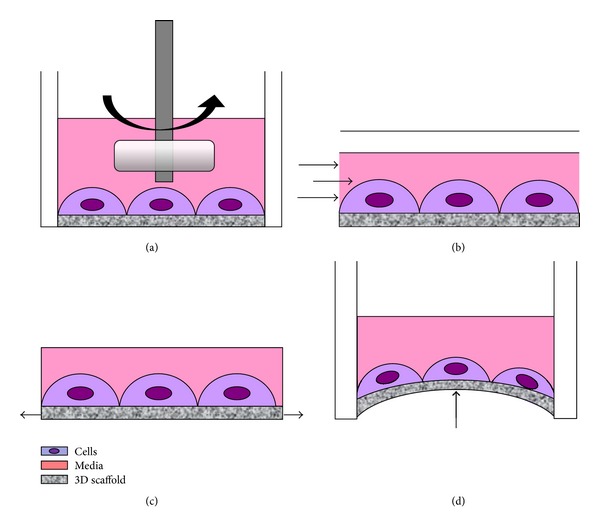
Schematic description of significant physical stimuli applied to cells growing in a bioreactor. A spinner flask/rotating vessel (a) improves nutrient and gas distribution by mixing culture media. Perfusion-based bioreactors (b) promote cell proliferation and matrix production via pulsatile flow and shear forces. Dynamic loading chambers (c, d) are intended to apply defined mechanical forces such as (cyclic) unidirectional (c) or biaxial (d) deformation to generate a strain.

In the last decade, the bioreactor technology in the field of cardiac tissue engineering evolved from very simple apparatus, such as spinner flask/rotating vessel (Figure 2(a)), to more complicated systems, such as perfusion bioreactors (Figure 2(b)) and dynamic loading chambers (Figures 2(c) and 2(d)). Older devices were intended for improving nutrient and gas distribution by mixing media without providing full control of culture parameters. These instruments became more and more sophisticated in order to apply defined physical stimuli [unidirectional or biaxial (cyclic) deformation, compression, stretch, perfusion, electrical force, etc.] appropriate to act on cell differentiation. A number of distinct configurations are nowadays available for specific purposes. Since the specific feature of cardiac muscle is the coordinated electromechanical coupling among its cells we here focus on an update on bioreactors used for engineering cardiac pseudotissue, reporting the approaches described in this field to date.

## 2. Bioreactors for Cardiac Tissue Engineering

The application of specific physical stimuli in a tailored bioreactor emerged as an appropriate strategy to obtain a bioengineered cardiac tissue, where mechanotransduction is known to play a significant role [37]. The initial evidence of effectiveness of mechanostimulation protocols in cardiac tissue engineering dates back to the second half of the nineties, when Vandenburgh et al. [38] showed that unidirectional mechanical stretch initiated *in vitro* a number of morphological alterations in a confluent cardiomyocyte population which were similar to those occurring during *in vivo* heart growth. A few years later, culturing engineered tissue under mixing of medium in a culture vessel was proven to help induce 3D constructs with cardiac-specific structural and electrophysiological properties [39, 40].

Since then a number of increasingly sophisticated approaches followed, and the purpose of this review is their listing according to the preferred approach endorsed for the application of the mechanostimulation protocol, that is, either the mechanical strain or the perfusion flow (see also Table 1).

### 2.1. Mechanical Strain

Six days of unidirectional stretch of engineered heart tissue (EHT)—made out of neonatal rat or embryonic chick cardiomyocytes mixed in collagen I—in a custom-made device improved their cellular organization and increased atrial natriuretic factor (ANF) mRNA and *α*-sarcomeric actin compared to unstretched controls [41]. The force of contraction of this EHT was up to fourfold higher after mechanical stimulation and the protocol was proposed as an *in vitro* model allowing morphological, molecular, and functional consequences of stretch to be studied under defined conditions. This same German research group developed shortly after [43] an improved technique to obtain circular EHT resulting in better technical feasibility, tissue homogeneity, and cardiomyocyte differentiation. After seven days of unidirectional cyclic stretch (10%; 2 Hz), the EHT displayed structural and functional features of a native differentiated myocardium and were proposed as a promising material for *in vitro* studies of cardiac function and tissue replacement therapy.

In the same year Akhyari et al. [42] proved that a mechanical stretch regimen—applied via the Biostretch apparatus (ICCT Technologies, Markham, ON, Canada) presented by Liu et al. [65] (mechanical properties and remodeling of hybrid cardiac constructs made from heart cells, fibrin, and a biodegradable, elastomeric knitted fabric were tested using the same apparatus by Boublik et al. in 2005 [46]) to human heart cells that were seeded on a 3D gelatin scaffold (Gelfoam sponge, Pharmacia & Upjohn Co., Kalamazoo, MI, USA)—improves the formation and enhances the strength of a bioengineered muscle graft. Dynamic stimulation of cells/gelatine constructs during 14 days (80 cycles/min with a 20% deformation of the initial length) generated a marked increase in cell proliferation, improved spatial cell distribution throughout the scaffold, and markedly increased the total amount of newly synthesized collagen matrix with fibres aligned in parallel to the axis of stress.

Iijima et al. [44] showed that mechanical load on skeletal muscle-derived cells is important for their transdifferentiation into the cardiac phenotype since passive cyclic stretch (60 cycles/min) of skeletal muscle-derived cells cocultured on silicone dishes with cardiomyocytes entirely restored the inhibition of their spontaneous beating produced with 5 *μ*M nifedipine. 

At this stage the application of a mechanostimulation protocol on terminally differentiated cells entered the age of majority: Zimmermann et al. [66, 67] provided the evidence that contractile cardiac tissue grafts, generated with the aid of mechanical strain *in vitro*, can survive after implantation and can support contractile function of infarcted rat hearts.

Since stem cells have entered the arena of regenerative medicine, the use of mechanostimulation protocols to address their cardiac differentiation was witnessed in several scientific reports. 

In 2008, Gwak et al. [50] investigated whether cyclic mechanical strain promotes cardiomyogenesis in mouse embryonic stem cell (ESCs) seeded on the elastic polymer poly(lactide-co-caprolactone) (PLCL). Mechanical load was applied in a custom-made bioreactor, previously described by Kim and Mooney [68]. The scaffolds were subjected to cyclic strain (10%; 1 Hz) in a standard incubator for 2 weeks. Mechanical load promoted cardiac-specific gene expression and tests *in vivo* showed a significant increase of grafting efficiency and the cardiomyogenic potential of the implanted cells.

In the same year, Shimko and Claycomb [51] used a bioreactor where ring-shaped constructs were stretched via a computer-controlled mechanism to explore the effects of long-term mechanical loading on mouse ESCs-derived cardiomyocytes. The cells, embedded in a 3D gelatinous scaffold (collagen type I and fibronectin), underwent cyclical mechanical stimulation (10%; 1, 2, and 3 Hz) for 3 days. This study demonstrated that ESCs-derived cardiomyocytes are actively responding to physical cues from the environment: *α*-cardiac actin, *α*-skeletal actin, *α*-myosin heavy chain (MHC), and *β*-MHC were all upregulated at 3 Hz. 

In 2009, Ge et al. [52] investigated cardiomyocyte differentiation potential of rat bone marrow-mesenchymal stem cells (BM-MSCs) treating these cells by applying 4% strain at 1 Hz. Biaxial mechanical stress, provided with a custom device previously described by Banes et al. [69], induced in BM-MSCs the expression of cardiomyocyte-specific genes including *α*-actin, connexin 43, *α*-MHC, and troponin I. 

During the most recent years, the application of mechanical cyclic strain to 2D cell cultures was often obtained using the Flexcell Strain Unit (Flexcell Int., Hillsborough, NC, USA), a commercial device where vacuum pressure applied to flexible-bottomed silicone culture plates produces uniaxial or biaxial deformation. 

In 2010, Salameh et al. [55] examined with this device whether cyclic mechanical stretch can affect localization of gap junctions with regard to the cell axis. Neonatal rat cardiomyocytes seeded on gelatin-coated membranes were stimulated (1 Hz; 0, 10, and 20% elongation) for 24 or 48 hours. Cyclic mechanical stretch (24 hour, 10%) induced elongation of the cardiomyocytes and orientation toward the stretch direction. Moreover, the distribution of connexin 43 and N-cadherin was accentuated at the cell poles. A significant increase in the transcription factors activator protein 1 and cAMP response element-binding protein was also scored.

In 2011, Maul et al. [60] used the Flexcell Unit for the systematic analysis of mechanical stimulation on SCs differentiation. Experiments were conducted using subconfluent MSCs for 5 days and demonstrated significant effects on morphology and proliferation, defining thresholds of cyclic stretch that potentiate (smooth) muscle protein expression. This systematic examination of the effects of mechanical stimulation on MSCs has implications for the understanding of SCs biology, as well as potential bioreactor designs for tissue engineering and cell therapy applications.

In the same year, Tulloch et al. [61] used human ESCs and induced pluripotent SC-derived cardiomyocytes in a 3-dimensional collagen matrix, to show that uniaxial mechanical stress conditioning—imparted with the Flexcell Unit—promotes a twofold increase of cardiomyocyte proliferation, matrix fiber alignment, and enhanced myofibrillogenesis and sarcomeric banding. Addition of endothelial cells enhanced cardiomyocyte proliferation, and addition of stromal supporting cells enhanced formation of vessel-like structures. These optimized human cardiac tissue constructs generate Starling curves, developing active force in response to increased resting length. Moreover, when transplanted onto hearts of athymic rats, the human myocardium survived and formed grafts closely apposed to host myocardium and containing human microvessels perfused by the host coronary circulation. The authors concluded that mechanical load and vascular cell coculture control cardiomyocyte proliferation, hypertrophy, and architecture of engineered human myocardium. Such constructs were proposed for studying human cardiac development as well as for regenerative therapy. 

To test the mechanical integrity and functionality of SCs engineered constructs prior to implantation, Hollweck et al. [57] proposed in 2011 a pulsatile bioreactor mimicking myocardial contraction. Mesenchymal stem cells derived from umbilical cord tissue (UCMSC) were colonized on titanium-coated polytetrafluorethylene (PFTE) scaffolds and underwent sinusoidal pulsation. Experiments to determine the adherence rate and morphology of UCMSC after mechanical loading showed an almost confluent cellular coating without damage on the cell surface and the bioreactor appeared an adequate tool for the mechanical stress of seeded scaffolds in order to precondition cardiac tissue engineered constructs *in vitro*.

All this evidence in the literature demonstrates the effect of cyclic mechanical stretch in maintaining, or addressing, a muscle phenotype. However, all the presented results were obtained using technical approaches useful for the experimental collection of proofs of principle but unlikely suitable for application in clinical protocols for regenerative medicine. Focusing on this issue, our group [62] designed a reliable innovative bioreactor, acting as a stand-alone cell culture incubator, easy to operate and effective in addressing rat MSCs seeded onto a 3D bioreabsorbable scaffold, toward a muscle phenotype via the transfer of a controlled and highly reproducible cyclic deformation. Electron microscopy, immunohistochemistry, and biochemical analysis of the pseudotissue constructs obtained after 1 week of cyclic mechanical stretch (10%; 1.6 Hz) showed cell multilayer organization and invasion of the 3D mesh of the scaffold. In addition they expressed typical markers of the muscle phenotype. This device is thus proposed as a prototypal instrument to obtain, using good manufacturing procedures, pseudotissue constructs to test in cardiovascular regenerative medicine.

### 2.2. Perfusion Bioreactors

Bioreactors used in cardiac tissue regeneration include devices where a mechanical load is transferred to the cells by culture medium routed (pulsatile flow, shear stress) through the construct with a perfusion loop.

In 2004, Radisic et al. [45] designed an *in vitro* culture system maintaining efficient oxygen supply to neonatal rat cardiomyocytes suspended in Matrigel and cultured on collagen sponges for 7 days with interstitial flow of medium. Constructs were assessed at timed intervals with respect to cell number, distribution, viability, metabolic activity, cell cycle, presence of contractile proteins (sarcomeric *α*-actin, troponin I, and tropomyosin), and contractile function in response to electrical stimulation. Perfusion resulted in higher cell viability and, in response to electrical stimulation, perfused constructs contracted synchronously and had a lower excitation threshold than controls. 

A microbioreactor array, fabricated using soft lithography and containing twelve independent microbioreactors perfused with culture medium, was presented by Figallo et al. [48] in 2007. This device enabled cultivation of cells either attached to substrates or encapsulated in hydrogels, at variable levels of hydrodynamic shear, and with automated image analysis of the expression of cell differentiation markers. This configuration was validated using primary rat cardiomyocytes and human ESCs evaluating correlations between the expression of smooth muscle actin and cell density for three different flow configurations.

Brown et al. [49] used a perfusion bioreactor suggesting that the provision of pulsatile interstitial medium flow to an engineered cardiac patch would result in enhanced tissue assembly by way of mechanical conditioning and improved mass transport. Cardiac patches, obtained by the seeding of neonatal rat cardiomyocytes onto Ultrafoam collagen hemostat discs, were cultured for 5 days subjected to two different overall flow rates (1.50 mL/min or 0.32 mL/min) at 1 Hz. The data reported in this study show that cultivation under pulsatile flow has beneficial effects on contractile properties and promotes cell hypertrophy.

In 2009, Hosseinkhani et al. [54] combined micro- and nanoscale technologies to fabricate a 3D collagen-poly (glycolic acid) (PGA) cell substrate for tissue engineering purposes where rat cardiac SCs (CSCs) were seeded and perfused to give a constant laminar flow of medium into the cell constructs to enhance their attachment and proliferation. Results demonstrated that this perfusion bioreactor improved the proliferation of CSCs *in vitro* compared with standard culture methods.

More recently, Kenar et al. [58] designed and developed a myocardial 3D patch formed by a microfibrous mat housing MSCs from human umbilical cord matrix (Wharton's Jelly) aligned in parallel to each other as they are in native myocardium. The 3D construct was dynamically cultured in a bioreactor by transiently perfusing cell medium through the macroporous tubing of the mat. After two weeks in the bioreactor, perfused cultures demonstrated enhanced cell viability, uniform cell distribution, and alignment due to nutrient provision from inside the 3D structure. 

This brief listing of flow-controlling devices for tissue cardiac engineering ends mentioning that in the same manuscript published by Maul et al. [60] in 2011, in addition to the use of the Flexcell Unit for the systematic analysis of mechanical stimulation on SCs differentiation (see above), the authors also evaluated on MSCs the impact of laminar shear stress—applied via the Streamer shear stress Flexcell device—that increased endothelial cell protein expression in a cell-contact-dependent manner. 

### 2.3. Hybrid Bioreactors

In a limited number of cases, the research in the field proposed hybrid devices, where more than a single type of mechanical stress is applied to cultured cells addressed toward the cardiac phenotype. As an example, electric and mechanical stimuli were occasionally applied to SCs on the hypothesis that the coupling of these stimuli might produce a synergy for their differentiation process.

Feng et al. [47] presented the first bioreactor system where cardiomyocytes from rat embryos were seeded on collagen-coated silicon membranes and cultured up to 4 days under electromechanical stimulation. Their results emphasized the importance of electrotensile forces on the augmentation of the contractile force in a “cardiac tissue equivalent.”

Barash et al. [53] used a custom-made electrical stimulator, integrated into a perfusion bioreactor originally described in 2006 by Dvir et al. [70], with the purpose of producing thick and functional cardiac patches. Rat ventricular cardiomyocytes were seeded on porous alginate scaffolds and subjected to a homogenous fluid flow regime with electrical stimulation. Results showed that the cultivation in this bioreactor for 4 days under perfusion and continuous electrical stimulus promoted cell elongation and striation. Immunostaining and western blotting analysis demonstrated that the expression level of connexin 43 was enhanced.

In 2011, Galie and Stegemann [56] validated a device where simultaneous mechanical and fluidic stress was applied to a 3D cell construct. Cardiac fibroblasts were suspended in a collagen type I gel to obtain a 3D cell construct and subjected to cyclic strain (5%; 1 Hz) and interstitial flow (10 mL/min). Cell viability was certified after 5 days in bioreactor under the application of these stimuli. This simple bioreactor system was thus proposed to model tissues such as the myocardium, which experiences interstitial fluid flow from perfusion through the extracellular matrix as well as cyclic strain from the systole—diastole cycle of the heart.

Kensah et al. [59] proposed in 2011 a multimodal bioreactor for mechanical stimulation and real-time direct measurement of contraction forces under continuous sterile culture conditions. The bioreactor's transparent cultivation chamber allows for microscopic assessment of tissue development. Additional functions include electric pacing of tissues, as well as the possibility to perfuse the central cultivation chamber allowing for continuous medium exchange and/or controlled addition of pharmacologically active agents. A functional bioartificial cardiac tissue was generated from rat cardiomyocytes in the bioreactor, where cyclic stretch induced cardiomyocyte hypertrophy and moderate increase in systolic force. Thus, this bioreactor is proposed as a tool for monitoring tissue development, and ultimately, optimizing SCs-based tissue replacement strategies in regenerative medicine.

Recently, Maidhof et al. [63] asserted that fixing the cell constructs in place for perfusion culture is a severe limitation. Thus, they proposed the design of a bioreactor to deliver simultaneous culture medium perfusion and electrical stimulation during the culture of engineered cardiac constructs free of external fixation. Neonatal rat heart cells were seeded onto channeled microporous elastomer poly(glycerol sebacate) (PGS) scaffolds and the neoformed pseudotissues were stimulated in the bioreactor for eight days. This dynamic culture protocol demonstrated significant improvement of DNA contents in the cell constructs, homogeneous cell distribution throughout the scaffold thickness, enhancement of cardiac protein expression (such as cardiac troponin T and connexin 43) and better cell morphology and overall tissue organization than observed in control group. Although cardiac cells did not form uniformly interconnected tissue after eight days of stimulation in the bioreactor, the results obtained in this study indicated that simultaneous perfusion and electric stimulation enhanced the development of engineered cardiac tissue. Thus, this device could be a relevant tool for cardiac repair *in vitro* studies and to understand the effective presence of a synergistic effect between perfusion and electric stimuli.

Usually, compression stimulus was extensively used for bone and cartilage tissue engineering [71–73]; however the favourable effect of mechanical compression was successfully certified in a cardiac tissue engineering protocol.

In particular, Shachar et al. [64] investigated how mechanical compression stimulus, combined with fluid shear stress provided by medium perfusion, could lead to the formation of a cardiac muscle tissue *in vitro*. Neonatal rat cardiac cells were seeded in Arginine-Glycine-Aspartate- (RGD-) attached alginate scaffolds and cultivated for 4 days in a bioreactor for compression and fluid flow stimulation. Two types of compression—intermittent (daily short-term 30 minutes) and continuous—were investigated. Upon application of the “intermittent compression” protocol, western blot showed enhancement of connexin 43, *α*-actinin, and N-cadherin expression.

## 3. Conclusions

Bioreactors appear as a key factor for a successful application of tissue engineering principles in cardiac regenerative medicine, where state-of-the-art mechanostimulation protocols have definitely proven to control cell proliferation, differentiation, and electrical coupling in engineered 3D cardiac tissue. A take-home message from this review of the current literature is that perfusion-based bioreactors are to be preferred when, for example, the cell function of interest is electively activated by shear stress, such as in the case of endothelial progenitor cells; when hydrogel/non-elastic scaffolds—which cannot be stretched by mechanical forces—are in use; or when scaffold nutrient diffusion has to be increased by a sustained flow. On the other hand, bioreactors producing mechanical deformation are more suitable with an elastomeric scaffold, where a coupled electromechanical stimulation appears effective in promoting elongation, striation, and acquisition of contractile force by the stretched cells.

Another issue emerging here is that the translation of these models into clinical products is not just around the corner. Rather than a scientific challenge, regulatory and commercial issues slow down the pace. Identifying a roadmap to drive this transition is an integral part of this challenge [74] where the compliance with regulatory guidelines and a robust and cost-effective approach have to match the already available encouraging proofs of principle. Governmental agencies in healthcare and stakeholders, together with clinical research societies and ethics committees, are now responsible for releasing guidelines and resources for the advancement expected by patients.

## Figures and Tables

**Table 1:** Bioreactor technology for cardiac tissue engineering.

Authors	Year	Cell source	Scaffold	Biophysical stimulus	Apparatus	Biological effects
Vandenburgh et al. [38]	1996	Neonatal rat cardiomyocytes	Collagen-coated silicon membranes	Unidirectional stretch	Mechanical cell stimulator	Increased foetal *β*- and adult *α*-MHC isoforms

Carrier et al. [40]	1999	Neonatal rat and embryonic chick cardiomyocytes	PGA	Perfusion by medium mixing	Rotating vessel microgravity bioreactor	Expression of cardiac-specific proteins and better ultrastructural organization

Fink et al. [41]	2000	Neonatal rat and embryonic chick cardiomyocytes	Collagen I	Unidirectional stretch	Custom stretching device	Improved organization of cardiomyocytes; hypertrophy

Akhyari et al. [42]	2002	Human heart cells (ventricular biopsy)	Gelfoam gelatine	Cyclic stretch	Biostretch	Enhancement of collagen matrix formation and organization

Zimmermann et al. [43]	2002	Neonatal rat cardiomyocytes	Collagen	Unidirectional cyclic stretch	Custom stretching device	Highly organized sarcomeres; adherens junctions, gap junctions, and desmosomes; well-developed T-tubular network; contractile characteristics of native myocardium

Iijima et al. [44]	2003	Rat cardiomyocytes and skeletal myocytes	Collagen-coated silicon membranes	Cyclic stretch	Mechanical cell stimulator	Expression of cardiac-specific proteins: cardiac troponin T, cadherin, and connexin 43

Radisic et al. [45]	2004	Neonatal rat cardiomyocytes	Ultrafoam collagen	Perfusion	Custom device	Thick, compact, and contractile cardiac constructs; higher cell viability

Boublik et al. [46]	2005	Rat heart cells	Hyaluronan	Cyclic stretch	Biostretch	Hybrid cardiac constructs with mechanical properties suitable for *in vitro *loading studies and *in vivo *implantation

Feng et al. [47]	2005	Neonatal rat cardiomyocytes	Silicon membranes	Mechanical stretch (+ electric field stimulation)	Custom stretching device	*In vitro* simulation of the electrical and mechanical responses of the myocardium *in vivo *

Figallo et al. [48]	2007	Neonatal rat cardiomyocytes and human ESCs	Collagen-coated glass	Perfusion	Custom microarray (MBA)	Increased smooth muscle actin and cell density

Brown et al. [49]	2008	Neonatal rat cardiomyocytes	Ultrafoam collagen	Perfusion	Custom device	Enhancement of contractile properties

Gwak et al. [50]	2008	Mouse ESC-derived cardiomyocytes	PLCL and PLGA	Cyclic stretch	Custom device	Enhancement of cardiac-specific gene expression: *α*-MHC, *α*-actin

Shimko and Claycomb [51]	2008	Mouse ESC-derived cardiomyocytes	Collagen I/fibronectin	Unidirectional cyclic stretch	Custom stretching device	Increased gene expression at 3 Hz cyclical stretch

Ge et al. [52]	2009	Rat BM-MSCs	Silicon membrane	Biaxial mechanical stretch	Custom stretching device	Expression of *α*-actin, connexion 43, *α*-MHC, and troponin I

Barash et al. [53]	2010	Neonatal rat cardiomyocytes	Alginate	Perfusion (+ electric field stimulation)	Custom device	Promotion of cell elongation and striation and enhancement of the expression level of connexin-43

Hosseinkhani et al. [54]	2010	Rat CSCs	Collagen-PGA nanofibers	Perfusion	Custom device	Significant enhancement of cell proliferation

Salameh et al. [55]	2010	Neonatal rat cardiomyocytes	Gelatin-coated silicone membrane	Biaxial mechanical stretch	Flexcell Tension System FX-4000	Self-organization of cardiomyocytes, enhanced connexin-43 expression and distribution at the cell poles

Galie and Stegemann [56]	2011	Rat cardiac fibroblasts	Collagen hydrogel	Mechanical stretch and interstitial flow	Custom device	Cell viability

Hollweck et al. [57]	2011	Human UCMSCs	PTFE	Biaxial mechanical stretch	Custom device	Confluent cellular coating without damage on the cell surface

Kenar et al. [58]	2011	Human MSCs (Wharton's Jelly)	PHBV-PLLA and PGS	Perfusion	Custom device	Enhanced cell viability, uniform cell distribution and alignment

Kensah et al. [59]	2011	Neonatal rat cardiomyocytes	Collagen I/Matrigel	Cyclic stretch and perfusion (+ electric field stimulation)	Custom device	Cardiomyocyte hypertrophy, shift of myosin heavy chain expression from the alpha to beta isoform

Maul et al. [60]	2011	Rat MSCs	Collagen-coated silicon membranes	Biaxial mechanical stretch (*∧*) or laminar shear stress (∗) or cyclic hydrostatic pressure (#)	(*∧*) Flexcell Tension System FX-4000 or (∗) Streamer shear stress Flexcell device or (#) custom device	Systematic examination of the effects of mechanical stimulation on MSCs

Tulloch et al. [61]	2011	Human ESCs and human induced pluripotent stem cell-derived cardiomyocytes	Collagen I	Cyclic stress	Flexcell Tension System FX-4000	Cardiomyocytes hypertrophy and proliferation

Govoni et al. [62]	2012	Rat MSCs	Hyaluronan	Unidirectional cyclic stretch	Custom device	Cell multilayer organization and invasion of the 3D mesh of the scaffold, muscle protein expression

Maidhof et al. [63]	2012	Rat heart cells	PGS	Perfusion (+ electric field stimulation)	Custom device	Improvement of DNA content, cell distribution throughout the scaffold thickness, cardiac protein expression, and cell morphology

Shachar et al. [64]	2012	Neonatal rat cardiomyocytes	RGD-attached alginate	Compression and shear stress	Custom device	Increased connexin-43, *α*-actin, and N-cadherin
